# Magnetic resonance imaging characteristics and survival outcomes of metaplastic breast carcinoma

**DOI:** 10.1371/journal.pone.0323541

**Published:** 2025-05-14

**Authors:** Wen-Pei Wu, Joseph Lin, Yu-Len Huang, Shou-Tung Chen, Chen-Te Chou, Dar-Ren Chen

**Affiliations:** 1 Department of Radiology, Changhua Christian Hospital, Changhua, Taiwan; 2 Department of Biomedical Imaging and Radiological Sciences, National Yang Ming Chiao Tung University, Taipei, Taiwan; 3 Division of General Surgery, Changhua Christian Hospital, Changhua, Taiwan; 4 Comprehensive Breast Cancer Center, Changhua Christian Hospital, Changhua, Taiwan; 5 Division of Breast Surgery, Yuanlin Christian Hospital, Yuanlin, Taiwan; 6 Department of Computer Science, Tunghai University, Taichung, Taiwan; The University of Queensland Faculty of Medicine, AUSTRALIA

## Abstract

**Objective:**

Metaplastic breast carcinoma is a rare type of breast carcinoma, and there are limited data about the magnetic resonance imaging (MRI) findings of metaplastic carcinoma. This study evaluates the MRI characteristics and prognostic outcomes across metaplastic carcinoma subtypes.

**Materials and Methods:**

In this retrospective cohort study, a total of 29 patients with histologically confirmed metaplastic carcinoma from 2011 to 2019 were enrolled. Clinical, pathological, and follow-up data, focusing on disease-free (DFS) and overall survival (OS), are recorded. Breast MRI findings were analyzed and categorized based on BI-RADS 5^th^ edition.

**Result:**

Among the participants, 19 had squamous carcinoma, 8 had metaplastic carcinoma with mesenchymal differentiation, and 2 had unclassified subtypes. The most common findings were a solitary mass (75.8%), high T2 signal (51.7%), and heterogenous enhancement (65.5%) with a washout kinetic curve (86.2%). The apparent diffusion coefficient (ADC) values in the metaplastic carcinoma with mesenchymal differentiation group were significantly higher (mean 1.83 +/- 0.50 x 10^-3^ mm^2^/s) in comparison with the squamous carcinoma group (mean 1.12 +/- 0.21 x 10^-3^ mm^2^/s). The cut-off point of the ADC value was 1.53x10^-3^ mm^2^/s, with a sensitivity of 71.4% and a specificity of 100% (AUC = 0.937). The five-year DFS and OS rates were 63% and 79% across the board, 78% and 89.5% in the squamous group, and 50% and 53% in the mesenchymal differentiation group, respectively,

**Conclusion:**

Metaplastic breast carcinoma is a diverse and aggressive malignancy with variable prognosis. Our findings indicate that mesenchymal differentiation is characterized by higher ADC values and correlates with a worse prognosis.

## Introduction

Metaplastic carcinoma is a rare subtype of breast cancer, accounting for 0.2–5% of all invasive breast carcinomas. Histologically, it is characterized by the presence of two or more malignant cell types, often a combination of epithelial and mesenchymal components, although a pure form with mesenchymal components can also exist. According to the most recent WHO classification, metaplastic carcinoma is categorized into several morphological subtypes, including low-grade adenosquamous carcinoma, fibromatosis-like metaplastic carcinoma, spindle cell carcinoma, squamous cell carcinoma, metaplastic carcinoma with heterologous mesenchymal differentiation, and mixed metaplastic carcinoma [1].

Due to the rarity of the metaplastic carcinoma, there are limited data correlating the imaging findings with clinical presentation, histopathology, and prognosis. Metaplastic breast carcinomas are known to exhibit distinct biological behaviors compared to ductal carcinomas of no special type [2]. They tend to present as larger tumors, are more likely to be triple-negative breast carcinomas (TNBC), exhibit resistance to chemotherapy, have a higher rate of hematogenous spread, and carry a poorer prognosis [3,4].

Early and accurate diagnosis of metaplastic carcinoma is crucial for survival. The rarity and lack of established imaging hallmarks make diagnosis a significant clinical challenge, as metaplastic carcinoma can be easily misidentified or overlooked in routine breast imaging [5–7]. On mammography, metaplastic carcinoma typically appears as an oval or round high-density mass with circumscribed or indistinct margins and usually lacks microcalcifications, features more suggestive of a benign lesion or a common fibrocystic change. Similarly, ultrasound often shows a solid, hypoechoic mass that may have a well-defined, lobulated outline, sometimes mimicking a fibroadenoma. Therefore, breast magnetic resonance imaging (MRI) has been used increasingly during the last decade due to its ability to reveal both the morphologic structure and the kinetic properties of the lesion [8,9]. Diffusion-weighted imaging (DWI) is a non-contrast MRI technique based on the free movement of water molecules in the extracellular space and reflects tissue cellularity [10,11]. The motion of water molecules is more restricted in tissues with high cellularity and less restricted in areas of low cellularity. Additionally, diffusion-weighted imaging (DWI) enables the quantitative assessment of water diffusion by utilizing the apparent diffusion coefficient (ADC). Breast cancer usually presents with a restricted diffusion of water molecules which leads to having a lower ADC value as compared to normal breast tissue and benign lesions of the breast [12,13].

Therefore, this retrospective study aims to assess the magnetic resonance imaging features of metaplastic carcinoma, evaluate surgical management and final pathological findings, and analyze the prognosis of metaplastic carcinoma over the past decade.

## Materials and methods

### Study population

The study was conducted in accordance with the Declaration of Helsinki (as revised in 2013). The institutional review board granted approval for this study (IRB No. 230102). Due to the retrospective nature of the data analysis, the IRB waived the requirement for signed informed consent. The data were accessed for research purposes on 2024/07/14. Throughout and after data collection, the authors had no access to any information that could identify individual participants.

**Fig 1:**
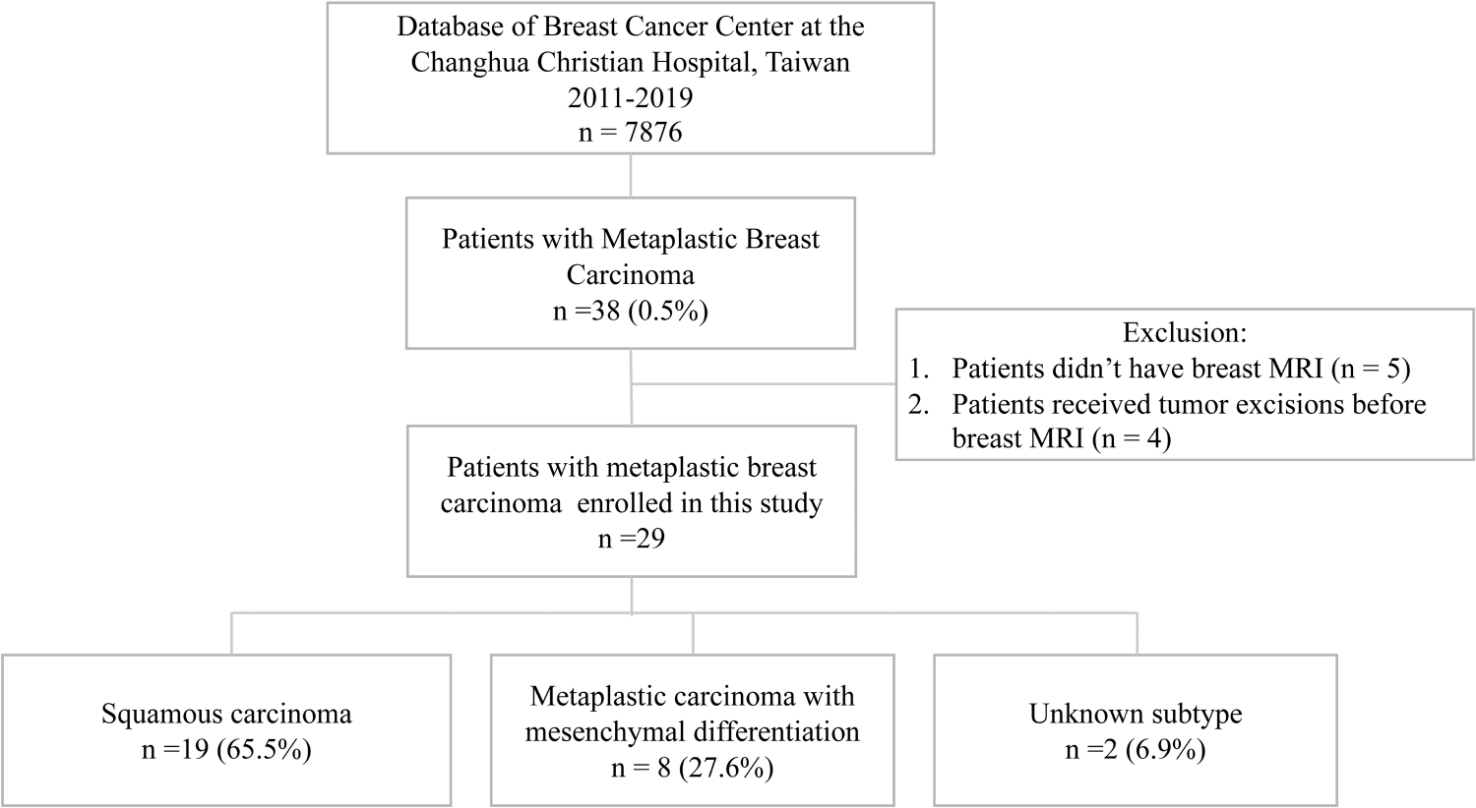
Flowchart of this study on metaplastic breast carcinoma. Out of a total database of 7,876 patients, 38 patients (0.5%) were identified with metaplastic breast carcinoma. Among these, 29 patients were enrolled in the study. The enrolled patients were then categorized into subtypes of metaplastic breast carcinoma: 19 patients (65.5%) with squamous carcinoma, 8 patients (27.6%) with carcinoma with mesenchymal differentiation, and 2 patients (6.9%) with an unknown subtype.

A retrospective review of 7876 histopathologically proven breast cancer cases diagnosed at our hospital between January 2011 and December 2019, revealed 38 cases (0.5%) of pathologically proven metaplastic breast carcinoma. The study excluded 5 patients who did not have a breast MRI and 4 patients who received tumor excisions prior to their breast MRI, resulting in 29 patients with metaplastic breast carcinoma being enrolled in the study (Fig 1).

The review included the collection of basic clinicopathologic information from the electronic medical record and the database of the cancer center, including patient age; body mass index; tumor type, grade, and size; biomarker profiles, including hormone receptor (HR) and human epidermal growth factor receptor 2 (HER2) status; Ki-67 proliferation index; disease free survival (DFS); overall survival (OS).

DFS is defined as the time from the date of the last surgical intervention until the onset of any disease recurrence, which includes both distant metastases and local recurrences, or death due to any cause. OS is the period from when the disease is first diagnosed to the occurrence of death from any cause.

### Pathological findings

Using hematoxylin and eosin (H&E) staining, the updated fifth edition [2019] of the World Health Organization (WHO) classification of breast tumors distinguishes MBC into six subtypes: low-grade adenosquamous carcinoma, fibromatosis-like metaplastic carcinoma, spindle cell carcinoma, squamous cell carcinoma, metaplastic carcinoma accompanied by heterologous mesenchymal differentiation, and mixed metaplastic carcinoma [1].

Using H&E staining along with immunohistochemistry (IHC) to assess HR status and HER2 status, which includes HER2 gene amplification evaluated by fluorescence in situ hybridization (FISH) and/or HER2 protein expression determined by IHC. The breast cancers in the study were classified into four distinct subtypes. These subtypes include HR-positive/HER2-negative, HR-positive/HER2-positive, HR-negative/HER2-positive, and HR-negative/HER2-negative, the latter being categorized as TNBC.

### MRI image acquisition and interpretation

All the patients received breast MRI within 2 weeks before operation. All breast MRI examinations were performed on a 3 Tesla MRI scanner (Verio, Siemens AG) with a 16-channel dedicated bilateral breast surface coil, and the MRI protocol was as follows: axial T1-weighted imaging (T1WI) and fat-suppressed T2-weighted imaging (T2WI) images; DWI imaging with a spin-echo, single-shot echo planar imaging sequence with three b values (b = 0, 400, 800 s/mm^2^); gadobutrol injection (Gadovist; Bayer Healthcare, Berlin, Germany) was used as the contrast enhancement agent at a dose of 0.1 ml/kg body weight and a rate of 2 ml/s, followed by an injection of saline; and axial 3D fat-saturated T1WI was performed after injection.

The breast MRI findings were retrospectively reviewed in consensus interpretations with 5^th^ American College of Radiology breast imaging reporting anad data system (ACR BI-RADS) lexicon by two board-certificated radiologists who were experienced in breast imaging (who had more than 15 years and 5 years experiences of breast MRI) [14]. The reviewers were blinded to patients’ clinicopathologic information, and final pathologic outcome. Measurement of tumor size by MRI and ADC value was based on using a commercially available MRI computer aid diagnosis (CAD) system with computer-based tumor segmentation by DynaCAD Version 2.1 (Philips Healthcare).

ADC values were obtained by manually placing several regions of interest (ROIs) within each lesion on the ADC map. ROIs were carefully drawn to encompass the most solid and cellular portions of the tumor, based on visual inspection of the ADC maps in conjunction with dynamic contrast-enhanced MRI (DCE-MRI) as a reference.

For each lesion, multiple ROIs were placed—typically on the slice showing the largest tumor cross-section—while avoiding areas of necrosis, cystic degeneration, and hemorrhage. Among the ROIs drawn, the one with the lowest ADC value was selected for analysis.

### Statistical analysis

Data analysis was performed using SPSS 22.0 software (IBM Corp., Chicago, IL, USA). Continuous variables were summarized using means with standard deviations or medians with ranges, as appropriate; categorical variables were presented as frequencies and percentages. Group comparisons for imaging and clinicopathologic characteristics were conducted using Chi-squared or Mann-Whitney tests. A receiver operating characteristic (ROC) curve was employed to determine the cut-off ADC value, along with corresponding sensitivity and specificity levels. DFS and OS were calculated with the Kaplan–Meier method, and groups were compared using the log-rank test. The Cox proportional hazards model was used to identify factors influencing OS and DFS. Statistical significance was defined as a two-tailed P-value of less than 0.05.

## Results

### Clinicopathological characteristics of the patient cohort

28 (96.6%) women and 1 (3.4%) man were enrolled in this study. The baseline clinical features and pathological subtypes of these patients are shown in Table 1. The median age of the patients was 56 years (range, 29–69 years). Tumor sizes measured on breast MRI showed that 5 (17.2%) had tumors smaller than 2 cm, 18 (62.1%) had tumors between 2–5 cm, and 6 (20.7%) had tumors larger than 5 cm. Pathologic lymph node metastases were negative in 19 (65.5%) of patients and positive in 10 (34.5%). The mean tumor size measured on MRI was 4.2 ± 2.4 cm, while the mean pathological size was 3.5 ± 2.0 cm, with MRI tending to slightly overestimate the tumor size. The intraclass correlation coefficient was 0.744, indicating moderate concordance between imaging and pathological measurements. The AJCC staging of the cancers varies, with 3 (10.3%) at stage I, 22 (75.9%) at stage II, 2 (6.9%) at stage III, and 2 (6.9%) at stage IV. Triple-negative breast cancer was present in 19 (65.5%) of the patients. Triple-negative breast cancer was present in 19 (65.5%) of the patients, while HR- and HER2 + and HR + and HER2- were both observed in 5(17.2%) of the cases. The Ki-67 proliferation index was high (≥20%) in 21 (72.4%) of patients, low (<20%) in 2 (6.8%), with 6 (20.7%) unknown.

**Table 1:** Clinical and pathologic characteristics of patients with metaplastic breast carcinoma.

Characteristics	N (%)
Gender	
Female	28 (96.6%)
Male	1 (3.4%)
Median Age at diagnosis (year)	56 (29-69)
Median follow-up period (mons)	27 (4-130)
BMI	
<25 kg/m2	22 (75.9%)
25-29 kg/m2	6 (20.7%)
>= 30 kg/m2	1 (3.4%)
Tumor size measured on breast MRI	
<2 cm	5 (17.2%)
2-5 cm	18 (62.1%)
>=5 cm	6 (20.7%)
Pathologic tumor size	
<=2 cm	3 (10.3%)
2-5 cm	21 (72.4%)
> 5 cm	5 (17.2%)
Pathologic lymph node metastases Negative	19 (65.5%)
Positive	10 (34.5%)
AJCC stage	
I	3 (10.3%)
II	22 (75.9%)
III	2 (6.9%)
IV	2 (6.9%)
Histologic subtypes	
Squamous cell carcinoma	19 (65.5%)
Metaplastic carcinoma with mesenchymal differentiation	8 (27.6%)
Unknown	2 (6.9%)
Triple-negative breast cancer	19 (65.5%)
Non-triple negative breast cancer	
HR + and Her-2+	0 (0%)
HR- and Her-2+	5 (17.2%)
HR + and Her-2-	5 (17.2%)
Ki-67	
Low (<20%)	2 (6.8%)
High (>=20%)	21 (72.4%)
Unknown	6 (20.7%)
Type of Surgery	
Breast conserving surgery	10 (34.5%)
Mastectomy	17 (58.6%)
Not done	2 (6.9%)

The histological features of metaplastic carcinomas are shown in Table 2. Nineteen cases (70.3%) had squamous differentiation, which was the most common subtype. 8 patients demonstrated mesenchymal components, which were subclassified according to their dominant histologic patterns.

**Table 2:** Results of pathologic review in patients with metaplastic breast carcinoma (n = 27).

Histologic features	N (%)
Squamous	19 (70.3%)
Osseous	2 (7.4%)
Matrix-producing	2 (7.4%)
Chondroid	1 (3.7%)
Sarcomatoid component	1 (3.7%)
Chondroid + spindle	1 (3.7%)
Chondroid + matrix producing	1 (3.7%)

Table 3 presents a comparison of clinical and pathologic characteristics between patients with squamous carcinoma (n = 19) and those with metaplastic carcinoma with mesenchymal differentiation (n = 8). Patients in the mesenchymal differentiation group tended to be younger at diagnosis (mean age 48.9 ± 9.2 years) than those in the squamous group (54.9 ± 11.2 years), though this difference was not statistically significant (*p* = 0.183). Tumor size was significantly larger in the mesenchymal differentiation group (5.2 ± 2.2 cm) compared to the squamous group (2.9 ± 1.3 cm; *p* = 0.002). No significant differences were observed in lymph node metastasis rates between the two groups (*p* = 0.824). Triple-negative breast cancer status was common in both groups, with 12 of 19 patients (63.2%) in the squamous group and 7 of 8 patients (87.5%) in the mesenchymal group (*p* = 0.206).

**Table 3:** Clinicopathologic characteristics of patients with squamous carcinoma and metaplastic carcinoma with mesenchymal differentiation.

	Squamous carcinoma (n = 19)	Metaplastic carcinoma with mesenchymal differentiation (n = 8)	P Value
Age at diagnosis (years)	54.9 ± 11.2	48.9 ± 9.2	0.183
Pathologic tumor size (cm)	2.9 ± 1.3	5.2 ± 2.2	0.002
Lymph nodes metastases			0.824
No	11	5	
Yes	8	3	
Triple negativity			0.206
No	7	1	
Yes	12	7	

### MRI Findings

**Fig 2:**
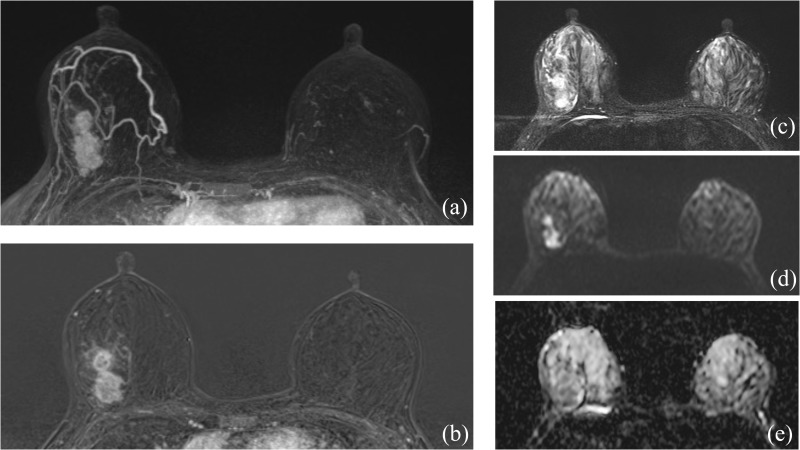
A 59-year-old female presented with a palpable mass in her right breast. After undergoing breast conserving surgery, the final pathology report revealed a grade III, ER-negative, PR-negative, and Her-2 positive squamous carcinoma. There were no axillary lymph node metastases identified. (1a-b) Breast MRI showed a dumbbell-shaped, heterogeneously enhancing mass with an irregular shape and margin, located in the upper outer quadrant of right breast. (1c) T2-weighted image showed iso- to hyper signal intensity of the tumor. (1d-e) the diffusion-weighted image showed hyperintensity and the corresponding ADC value showed 1.49x10^-3^ mm^2^/s.

**Fig 3:**
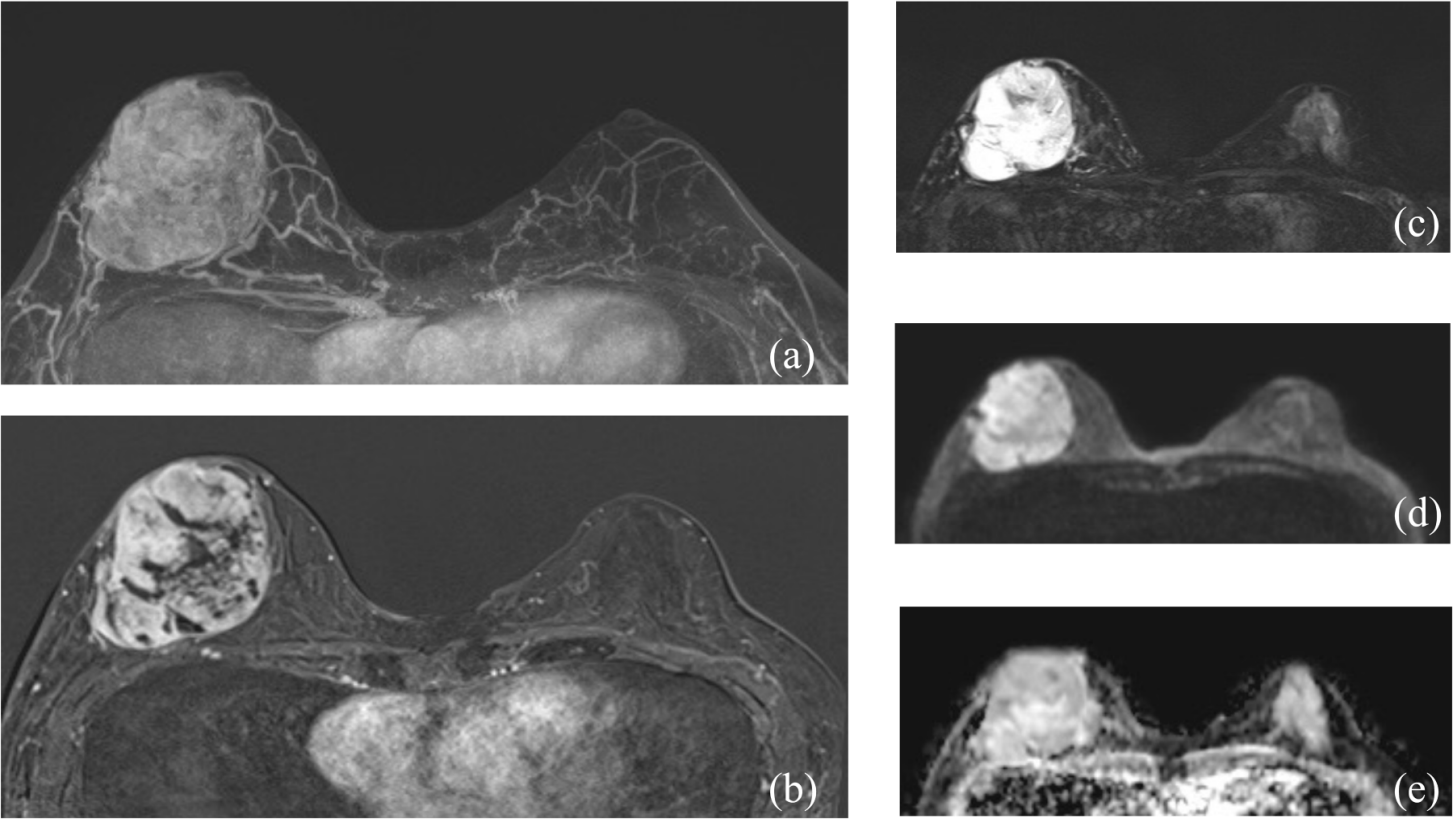
MRI findings in a 50-year-old female with metaplastic carcinoma with mesenchymal differentiation. (a-b) Axial Maximum Intensity Projection (MIP) and subtraction T1-weighted contrast-enhanced images display a large, round mass in the right breast, exhibiting heterogeneous enhancement and a circumscribed margin. (c) The axial T2-weighted image shows the mass with high signal intensity. (d-e) The diffusion-weighted imaging (DWI) and the corresponding Apparent Diffusion Coefficient (ADC) map reveal a high ADC value of 2.04 x 10^-3^ mm²/s.

Tumors in patients with metaplastic carcinoma with mesenchymal differentiation were significantly larger (6.1 ± 3.1 cm) compared to those in patients with squamous carcinoma (3.6 ± 1.7 cm), with statistical significance (P = 0.034). Multifocality (having multiple foci) was not observed in the mesenchymal differentiation group, contrasting with 26.3% in the squamous carcinoma group, although this difference was not statistically significant (P = 0.280). The morphology was predominantly mass-type in both groups, with a significant presence in the mesenchymal differentiation group (100% vs. 73.7%). Shape and margins were equally distributed between irregular and round, and circumscribed and irregular, respectively, in the mesenchymal differentiation group, showing no significant difference from the squamous carcinoma group. Kinetic curve assessments in both initial and delayed phases did not significantly differ between the two groups, with most tumors showing fast initial phase kinetics and washout in the delayed phase. (As seen in Table 4, Figs 2–3)

**Table 4:** MRI findings of patients with metaplastic breast carcinoma.

Characteristics	Squamous carcinoma (n = 19)	Metaplastic carcinoma with mesenchymal differentiation (n = 8)	*P* value
Size (cm)	3.6 + /- 1.7	6.1 + /-3.1	0.034
Multifocality			0.280
MF or MC	5 (26.3%)	0 (0%)	
Single	14 (73.7%)	8 (100%)	
Morphology			0.280
Mass	14 (73.7%)	8 (100%)	
Mass and NME	5 (26.3%)	0 (0%)	
T2 signal			0.187
Hyper	9 (47.4%)	6 (75.0%)	
Iso- to hyper	10 (52.6%)	2 (25.0%)	
Peritumor edema			0.133
Yes	15 (78.9%)	4 (50.0%)	
None	4 (21.1%)	4 (50.0%)	
Internal enhancement			0.029
Heterogenous	11 (57.9%)	8 (100%)	
Rim	8 (42.1%)	0 (0%)	
Shape			0.516
Irregular	8 (42.1%)	4 (50%)	
Round	11 (57.9%)	4 (50%)	
Margins			0.516
Circumscribed	8 (42.1%)	4 (50%)	
Irregular	11 (57.9%)	4 (50%)	
Kinetic curve (Initial phase)			1.000
Fast	16 (84.2%)	7 (87.5%)	
Medium	3 (15.8%)	1 (12.5%)	
Kinetic curve (Delayed phase)			1.000
Plateau	2 (10.5%)	0 (0%)	
Washout	17 (89.5%)	8 (100%)	
ADC value	1.12 + /-0.21	1.83 + /-0.50	0.001

MF, multifocal; MC, multicentric; NME, non-mass enhancement; ADC, apparent diffusion coefficient.

**Fig 4:**
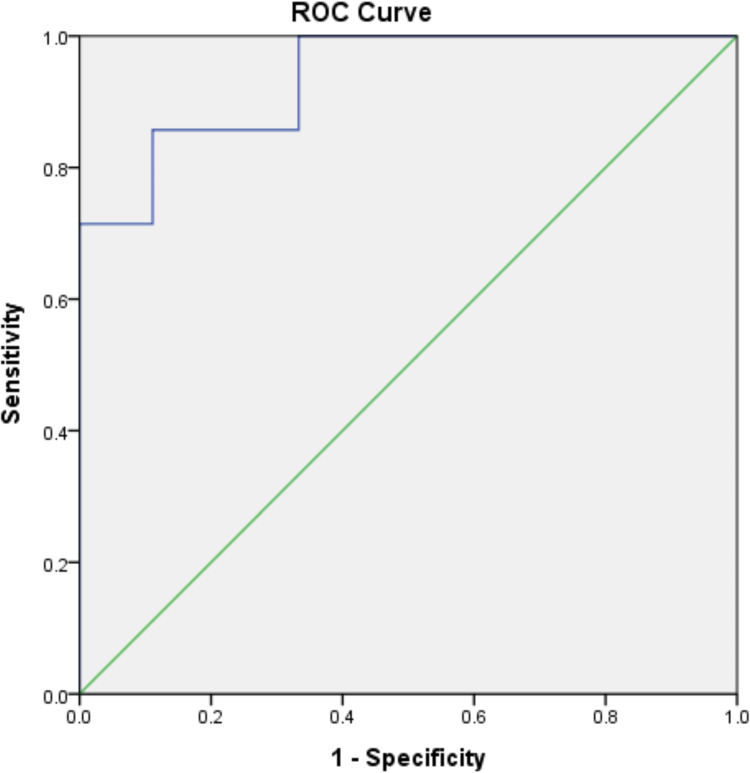
Receiver operating characteristic curve for distinguishing metaplastic carcinoma with mesenchymal differentiation from squamous carcinoma on ADC value, with the area under the curve (0.937, 95% confidence interval: 0.833 to 1.000).

ADC values were significantly higher in the mesenchymal differentiation group (1.83 ± 0.50 x10^-3^ mm^2^/s) compared to the squamous carcinoma group (1.12 ± 0.21 x10^-3^ mm^2^/s), indicating a statistical significance (P = 0.001). The analysis of ROC curve determined ADC>= 1.53x10^-3^ mm^2^/s as the optimum cut-off value distinguishing metaplastic carcinoma with mesenchymal differentiation group and squamous carcinoma group, with a sensitivity of 71.4%, a specificity of 100%, and a very high diagnostic accuracy (AUC = 0.937, 95%CI: 0.833 to 1.000), as seen in Fig 4.

### Overall survival

The mean OS was determined using Kaplan-Meier curves, with an overall duration of 102.8 ± 10.6 months. For specific histology types, squamous cell carcinoma showed a mean survival of 117.8 ± 8.6 months, while metaplastic carcinoma with mesenchymal differentiation exhibited a lower mean survival of 69.5 ± 24.8 months. The 5-year OS rate was 79% in the overall group, while 89.5% in the squamous cell carcinoma group and 53% in the metaplastic carcinoma with mesenchymal differentiation group and the hazard ratio (HR) of 1.79, though the difference was insignificant (P = 0.181).

**Fig 5:**
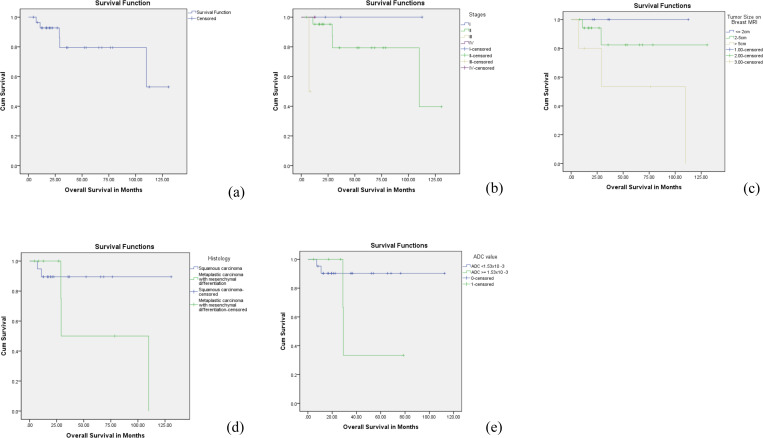
Kaplan-Meier overall survival (OS) outcomes for patients with metaplastic breast carcinoma. (a) the OS curve for the study cohort. (b) the OS curve stratified by American Joint Committee on Cancer (AJCC) tumor stages. (c) the OS curve based on tumor size on the breast MRI. (d) the OS curve with different histological types of metaplastic breast carcinoma: squamous carcinoma versus metaplastic carcinoma with mesenchymal differentiation. (e) the OS curve in relation to apparent diffusion coefficient (ADC) values, using a cut-off value of 1.53x10^-3^ mm²/s.

Considering the ADC cutoff value of 1.53x10^-3^ mm2/s, the mean overall survival for those with a lower ADC value was 102.5 ± 6.8 months, compared to 45.5 ± 13.5 months for those with a higher ADC value. The survival outcomes demonstrate statistically significant variations across different tumor stages, with survival rates declining as the disease progresses from stage I to the more advanced stages III and IV (P = 0.003). These findings are seen in Fig 5.

The Cox regression analysis for patients’ OS is presented in Table 5. Tumor size measured on breast MRI was associated with increased probability of death (HR 4.77, CI 0.933–24.390, p = 0.043). No other risk factor was significantly related to patients’ OS.

**Table 5:** Cox regression analysis for Overall Survival (OS) in patients with metaplastic breast carcinoma.

		Cox Regression Analysis	
Patient Characteristic	HR	95% CI	P value
Age	0.988	0.902-1.083	0.802
BMI	1.302	0.312-5.444	0.717
Pathologic tumor size	4.563	0.812-25.659	0.085
Lymph node metastases	1.461	0.205-10.390	0.703
Histology	3.256	0.5287-20.081	0.181
Ki-67 > 20%	25.261	0.000-6592969.069	0.427
Tumor size on breast MRI	4.77	0.933-24.390	0.043
Multifocality on breast MRI	0.031	0.000-415.716	0.234
ADC value	3.832	0.537-27.361	0.180

HR: hazard ratio, CI: confidence interval, BMI: body mass index, ADC: apparent diffusion coefficient.

### Disease-free survival

**Fig 6:**
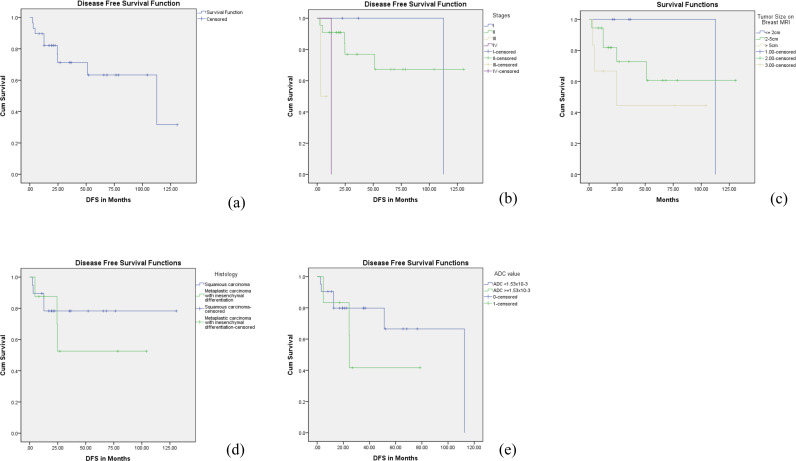
Kaplan-Meier disease-free survival (DFS) outcomes for patients with metaplastic breast carcinoma. (a) the DFS curve for the study cohort. (b) the DFS curve stratified by American Joint Committee on Cancer (AJCC) tumor stages. (c) the DFS curve based on tumor size on the breast MRI. (d) the DFS curve with different histological types of metaplastic breast carcinoma: squamous carcinoma versus metaplastic carcinoma with mesenchymal differentiation. (e) the DFS curve in relation to apparent diffusion coefficient (ADC) values, using a cut-off value of 1.53x10^-3^ mm²/s.

Similarly, the DFS as estimated using Kaplan-Meier curves, was approximately 85.0 ± 11.5 months overall. Squamous cell carcinoma exhibited a longer mean disease-free time of 104.0 ± 11.8 months, in contrast to metaplastic carcinoma with mesenchymal differentiation, which had a significantly shorter mean disease-free time of 63.9 ± 17.2 months, although this difference was not statistically significant (P = 0.504). The 5-year DFS was 63% in the overall patient group, while 78% in the squamous cell carcinoma group and 50% in the metaplastic carcinoma with mesenchymal differentiation group. These findings are seen in Fig 6. The Cox regression analysis for patients’ disease-free survival is presented in Table 6. Increasing age is associated with a slight decrease in the risk of disease recurrence or progression, and this result is statistically significant, with the hazard ratio is 0.919 (P = 0.035).

**Table 6:** Cox regression analysis for Disease-Free Survival (DFS) in patients with metaplastic breast carcinoma.

		Cox Regression Analysis	
Patient Characteristic	HR	95% CI	P value
Age	0.919	0.858-0.984	0.035
BMI	0.644	0.157-2.643	0.541
Pathologic tumor size	2.077	0.546 -7.906	0.287
Lymph node metastases	2.291	0.544-9.642	0.258
Histology	1.656	0.369-7.434	0.511
Ki-67 > 20%	0.346	0.062-1.914	0.224
Tumor size on breast MRI	2.188	0.713-6.716	0.171
Multifocality on breast MRI	1.908	0.454-8.010	0.378
ADC value	2.014	0.477-8.512	0.341

HR: hazard ratio, CI: confidence interval, BMI: body mass index, ADC: apparent diffusion coefficient.

When considering the ADC cutoff value of 1.53x10^-3 mm²/s, patients with a lower ADC value recorded a mean disease-free time of 83.3 ± 12.4 months, compared to 43.8 ± 13.3 months for those with a higher ADC value.

Specifically, Stage I disease showed a mean disease-free survival of 112 ± 0 months, Stage II disease had 96.6 ± 12.9 months, and Stage III disease significantly dropped to 5.6 ± 1.8 months (P = 0.006). This highlights the critical impact of tumor stage on disease progression and patient outcomes.

## Discussion

Metaplastic carcinoma of the breast is a rare subtype, accounting for approximately 0.5% of all breast cancer cases at our institution. This rate aligns with other studies, which report an incidence of 0.5% in the United States and 0.6% in Korea, with reported incidence rates ranging from 0.5% to 1.9% of all breast cancers globally [4,15,16]. It is characterized by poorly differentiated tumors with a heterogeneous histology, containing both mesenchymal and epithelial elements.(3) Metaplastic carcinoma is known for its clinical aggressiveness and generally poor prognosis. However, despite its aggressive behavior, metaplastic carcinoma often exhibits benign features on standard imaging methods like mammography and ultrasonography [17–19]. Breast MRI has emerged as a promising tool to better differentiate metaplastic carcinoma from benign breast lesions [20,21]. To investigate this further, we conducted a retrospective analysis examining breast MRI findings and assessing the prognostic characteristics of patients with metaplastic carcinoma.

In this study, metaplastic carcinoma primarily affects middle-aged women, with a median age of 56 years (range, 29–69) and with the average longest diameter of 4.2 + /- 2.4 cm. Consistent with previously reported studies, axillary node involvement is less common in these tumors due to their tendency to spread hematogenously rather than through the lymphatic system, with rates between 25–40% [22,23]. In our series, 10 (34.5%) of the patients had metastatic axillary lymph nodes. The triple negative rate of metaplastic breast carcinoma in clinical studies is reported to be between 48% and 90.0% [24–26]. In our study, 19(65.5%) patients were found to have triple negative tumors. This high prevalence of triple negativity is consistent with previous findings, indicating that metaplastic carcinoma typically presents with more aggressive characteristics compared to other breast cancer subtypes.

Squamous carcinoma represented two-thirds of the metaplastic carcinoma cases, while the remaining one-third exhibited mesenchymal differentiation. Compared with squamous cell carcinoma, metaplastic carcinoma with mesenchymal differentiation typically presents larger tumors at diagnosis. Yao et al. found that the most prevalent subtypes of metaplastic carcinoma were squamous carcinoma (52.8%) and metaplastic carcinoma with mesenchymal differentiation (30.6%), a distribution similar to that of our cohort [17]. However, earlier studies have demonstrated variations in the distribution of these subtypes [3].

Of increasing interest is the role of breast MRI on the subtypes of metaplastic carcinoma. The mesenchymal differentiation subtype tends to present with a mass with T2 hypersignal intensity, heterogenous enhancement with a washout kinetic curve. In our study, internal enhancement of the tumors with rim enhancement is only seen in squamous carcinoma. This finding is also seen in the previous reported studies. The cystic part within the tumor is commonly associated with squamous component [23,27].

The ADC values showed a significant difference (P = 0.001) between squamous carcinoma and metaplastic carcinoma with mesenchymal differentiation. In the group with mesenchymal differentiation, they were significantly higher (mean 1.83 ± 0.50 × 10 − 3 mm2/s) in comparison with the group with squamous carcinoma (mean 1.12 ± 0.21 × 10 − 3 mm2/s). Gladys et al. reported that the optimum threshold ADC value for diagnosing benign and malignant breast tumors was 1.21 × 10^−3^ mm^2^/s. The ADC value, a measurable diffusion-weighted imaging parameter of the Brownian motion of water, is related to cellular density, tumor grade, tumor subtype, Ki-67 index, and cell apoptosis [28,29]. Chen et al. have reported significant differences in the ADC values between mesenchymal-like and epithelial-like tumors in mice [30]. Their findings indicate that mesenchymal-like tumors exhibit higher ADC values, while epithelial-like tumors display lower ADC values. Metaplastic carcinoma with mesenchymal differentiation, is often termed ‘matrix-producing carcinoma’ due to its composition, which includes a mix of heterologous mesenchymal elements [31]. These elements span from chondroid to osseous differentiation and may be the cause of higher ADC values. Prior literature provides limited data on ADC values in metaplastic carcinoma [17,27]. The published studies on diffusion-weighted MRI of breast lesions do not differentiate between the various rare subtypes of metaplastic carcinomas, due to the rarity and heterogeneity of these tumors. We demonstrate a statistically significant difference in ADC values specifically in metaplastic carcinoma with mesenchymal differentiation. We further underscore that using an optimal ADC cutoff in our analysis allowed us to distinguish lesions with mesenchymal differentiation with high diagnostic performance. Therefore, our novel finding may also have prognostic implications: if diffusion MRI can suggest mesenchymal differentiation, it might help inform prognosis and management decisions earlier (for example, by recognizing a tumor subtype that tends to behave more aggressively).

Due to the increased likelihood of distant metastases and poor response to chemotherapy, metaplastic carcinoma has a worse prognosis than other types of breast cancer [4,32,33]. The 5-year DFS and OS rates for patients with metaplastic carcinomas were 63% and 79% in our study. The study by Cimino-Mathews et al found a 5-year DFS of 64% and a 5-year OS rate of 69% among 45 patients with metaplastic carcinoma, which is similar to the results of our study [34]. In our study, the 5-year DFS and OS in the squamous carcinoma and metaplastic carcinoma with mesenchymal differentiation are 78%, 89.5%, and 50%, 53%, respectively, though no statistical significance. Our findings align with the study by Tan et al., which indicated that mesenchymal differentiation is associated with poorer survival compared to other subtypes [35]. However, Rakha et. al reported that spindle and mixed spindle and squamous were associated with the worst prognosis [36]. Conversely, Tadros et. al reported that the heterologous mesenchymal subtype is associated with the best survival, whereas the squamous subtype is associated with the worst survival [37]. These conflicting findings regarding survival rates across subtypes may reflect variations in survival endpoints, study populations, or the overall heterogeneity of metaplastic carcinomas. Further, larger studies are required to clarify these discrepancies.

First, the small sample size and single-center design limit the statistical power of the study and reduce the reliability of subgroup analyses. Although metaplastic breast carcinoma comprises a diverse spectrum of histologic subtypes, our analysis included only squamous carcinoma and mesenchymal differentiation, which limits the study’s comprehensiveness. Second, the retrospective nature of the study inherently introduces selection and measurement biases. Additionally, the lack of molecular and genetic profiling, along with the absence of detailed treatment information, restricts our ability to investigate tumor heterogeneity and evaluate the effects of specific therapeutic interventions. Despite these limitations, this study remains one of the largest single-center cohorts examining metaplastic breast carcinoma with comprehensive imaging data and extended follow-up. Future multi-institutional studies with larger, more diverse populations and integrated clinical, molecular, and treatment data are warranted to validate and expand upon our findings.

Metaplastic breast carcinoma in the Taiwan presents a heterogenous disease encompassing biologically different tumor classes with comparable overall and disease-free survival. Breast MRI can provide valuable diagnostic information for metaplastic carcinoma, while metaplastic carcinoma with mesenchymal differentiation exhibits a higher ADC value. The tumor histology components and molecular alterations have led to the water diffusion restriction. There is a need for a multi-institutional prospective study with detailed imaging and pathological analysis with longer follow up period for identifying definitive prognostic and predictive factors.

## References

[1] Tan PH, Ellis I, Allison K, Brogi E, Fox SB, Lakhani S, et al. The 2019 World Health Organization classification of tumours of the breast. Histopathology. 2020;77(2):181–5. doi: 10.1111/his.14091 32056259

[2] Zhao S, Ma D, Xiao Y, Jiang Y-Z, Shao Z-M. Clinicopathologic features and prognoses of different histologic types of triple-negative breast cancer: a large population-based analysis. Eur J Surg Oncol. 2018;44(4):420–8. doi: 10.1016/j.ejso.2017.11.027 29429597

[3] Thomas HR, Hu B, Boyraz B, Johnson A, Bossuyt VI, Spring L, et al. Metaplastic breast cancer: a review. Crit Rev Oncol Hematol. 2023;182:103924. 36696934 10.1016/j.critrevonc.2023.103924

[4] Thomas A, Douglas E, Reis-Filho JS, Gurcan MN, Wen HY. Metaplastic breast cancer: current understanding and future directions. Clin Breast Cancer. 2023;23(8):775–83. doi: 10.1016/j.clbc.2023.04.004 37179225 PMC10584986

[5] Patterson SK, Tworek JA, Roubidoux MA, Helvie MA, Oberman HA. Metaplastic carcinoma of the breast: mammographic appearance with pathologic correlation. AJR Am J Roentgenol. 1997;169(3):709–12. doi: 10.2214/ajr.169.3.9275883 9275883

[6] Jia Y, He C, Liu L, Sun L, Chen Y, Luo Y, et al. A retrospective study of the imaging and pathological features of metaplastic breast carcinoma and review of the literature. Med Sci Monit. 2019;25:248–58. doi: 10.12659/MSM.912107 30618455 PMC6338010

[7] Langlands F, Cornford E, Rakha E, Dall B, Gutteridge E, Dodwell D, et al. Imaging overview of metaplastic carcinomas of the breast: a large study of 71 cases. Br J Radiol. 2016;89(1064):20140644. doi: 10.1259/bjr.20140644 27245135 PMC5124866

[8] Mota BS, Reis YN, de Barros N, Cardoso NP, Mota RMS, Shimizu C, et al. Effects of preoperative magnetic resonance image on survival rates and surgical planning in breast cancer conservative surgery: randomized controlled trial (BREAST-MRI trial). Breast Cancer Res Treat. 2023;198(3):447–61. doi: 10.1007/s10549-023-06884-5 36786946 PMC10036439

[9] Lafcı O, Celepli P, Seher Öztekin P, Koşar PN. DCE-MRI radiomics analysis in differentiating luminal A and luminal B breast cancer molecular subtypes. Acad Radiol. 2023;30(1):22–9. doi: 10.1016/j.acra.2022.04.004 35595629

[10] Whisenant JG, Romanoff J, Rahbar H, Kitsch AE, Harvey SM, Moy L, et al. Factors affecting image quality and lesion evaluability in breast diffusion-weighted MRI: observations from the ECOG-ACRIN Cancer Research Group Multisite Trial (A6702). J Breast Imaging. 2020;3(1):44–56. doi: 10.1093/jbi/wbaa103 33543122 PMC7835633

[11] Lee SH, Shin HJ, Moon WK. Diffusion-weighted magnetic resonance imaging of the breast: standardization of image acquisition and interpretation. Korean J Radiol. 2021;22(1):9–22. doi: 10.3348/kjr.2020.0093 32901461 PMC7772373

[12] Tsvetkova S, Doykova K, Vasilska A, Sapunarova K, Doykov D, Andonov V, et al. Differentiation of benign and malignant breast lesions using ADC values and ADC ratio in breast MRI. Diagnostics (Basel). 2022;12(2):332. doi: 10.3390/diagnostics12020332 35204423 PMC8871288

[13] Caivano R, Villonio A, D’ Antuono F, Gioioso M, Rabasco P, Iannelli G, et al. Diffusion weighted imaging and apparent diffusion coefficient in 3 tesla magnetic resonance imaging of breast lesions. Cancer Invest. 2015;33(5):159–64. doi: 10.3109/07357907.2015.1019674 25831024

[14] Radiology A, D’Orsi C. ACR BI-RADS atlas: breast imaging reporting and data system; mammography, ultrasound, magnetic resonance imaging, follow-up and outcome monitoring, data dictionary. ACR, American College of Radiology; 2013.

[15] Mills MN, Yang GQ, Oliver DE, Liveringhouse CL, Ahmed KA, Orman AG, et al. Histologic heterogeneity of triple negative breast cancer: A National Cancer Centre Database analysis. Eur J Cancer. 2018;98:48–58. doi: 10.1016/j.ejca.2018.04.011 29870876

[16] Jung S-Y, Jung K-W, Ha J, Won Y-J, Kim YA, Kwon Y, et al. Different patterns of conditional survival of breast cancer patients by age and histologic types: evidence from the korean nationwide registry. Cancer Epidemiol Biomarkers Prev. 2019;28(7):1169–76. doi: 10.1158/1055-9965.EPI-18-1072 31028082

[17] Yao MX, Li L, Ye WT, Liu Y, Wang Y, Zhu W, et al. Multimodal imaging features and prognosis of metaplastic breast carcinoma. Acad Radiol. 2023. 10.1016/j.acra.2023.10.017 38030514

[18] Yao M, Luo S, Li L, Wang Y, Zhu W, Liu Y, et al. Sonographic and clinicopathologic features of metaplastic breast carcinoma and infiltrating ductal carcinoma: a comparative single-center cohort study. Quant Imaging Med Surg. 2024;14(1):909–19. doi: 10.21037/qims-23-1096 38223107 PMC10784059

[19] Püsküllüoğlu M, Świderska K, Konieczna A, Rudnicki W, Pacholczak-Madej R, Kunkiel M, et al. Discrepancy between tumor size assessed by full-field digital mammography or Ultrasonography (cT) and Pathology (pT) in a multicenter series of breast metaplastic carcinoma patients. Cancers (Basel). 2023;16(1):188. doi: 10.3390/cancers16010188 38201615 PMC10778481

[20] Mann RM, Cho N, Moy L. Breast MRI: state of the art. Radiology. 2019;292(3):520–36. doi: 10.1148/radiol.2019182947 31361209

[21] Scaranelo AM What’s hot in breast MRI. Can Assoc Radiol J. 2022;73(1):125–40. 34384041 10.1177/08465371211030944

[22] Chhieng C, Cranor M, Lesser ME, Rosen PP. Metaplastic carcinoma of the breast with osteocartilaginous heterologous elements. Am J Surg Pathol. 1998;22(2):188–94. doi: 10.1097/00000478-199802000-00006 9500219

[23] Velasco M, Santamaría G, Ganau S, Farrús B, Zanón G, Romagosa C, et al. MRI of metaplastic carcinoma of the breast. AJR Am J Roentgenol. 2005;184(4):1274–8. doi: 10.2214/ajr.184.4.01841274 15788609

[24] Takala S, Heikkilä P, Nevanlinna H, Blomqvist C, Mattson J. Metaplastic carcinoma of the breast: Prognosis and response to systemic treatment in metastatic disease. Breast J. 2019;25(3):418–24. doi: 10.1111/tbj.13234 30925636

[25] Bicky T, Salome A, Bernadette AC, Jiao X, Hamed D, Ye C, et al. Metaplastic breast cancer: characteristics and survival outcomes. Cureus. 2022;14. 10.7759/cureus.28551 PMC9517584 36185859

[26] Metaplastic breast carcinoma: sonographic and clinicopathologic comparison with infiltrating ductal carcinoma. 2023.

[27] Wang S, Lou J, Zou Q, Jiang Y, Wang S, Shi H. Metaplastic carcinoma of the breast: MRI features with clinical and histopathologic correlation. Acad Radiol. 2023;30(9):1786–93. 36137916 10.1016/j.acra.2022.08.026

[28] Wang Y, Chen ZE, Yaghmai V, Nikolaidis P, McCarthy RJ, Merrick L, et al. Diffusion-weighted MR imaging in pancreatic endocrine tumors correlated with histopathologic characteristics. J Magn Reson Imaging. 2011;33(5):1071–9. doi: 10.1002/jmri.22541 21509863

[29] Surov A, Clauser P, Chang Y-W, Li L, Martincich L, Partridge SC, et al. Can diffusion-weighted imaging predict tumor grade and expression of Ki-67 in breast cancer? A multicenter analysis. Breast Cancer Res. 2018;20(1):58. doi: 10.1186/s13058-018-0991-1 29921323 PMC6011203

[30] Chen Y-W, Pan H-B, Tseng H-H, Chu H-C, Hung Y-T, Yen Y-C, et al. Differentiated epithelial- and mesenchymal-like phenotypes in subcutaneous mouse xenografts using diffusion weighted-magnetic resonance imaging. Int J Mol Sci. 2013;14(11):21943–59. doi: 10.3390/ijms141121943 24196357 PMC3856043

[31] Xu D, Hou L. Clinicopathologic characteristics of mixed epithelial/mesenchymal metaplastic breast carcinoma (carcinosarcoma): a meta-analysis of Chinese patients. Pol J Pathol. 2019;70(3):174–82. 31820860 10.5114/pjp.2019.90393

[32] Ong CT, Campbell BM, Thomas SM, Greenup RA, Plichta JK, Rosenberger LH, et al. Metaplastic breast cancer treatment and outcomes in 2500 patients: a retrospective analysis of a national oncology database. Ann Surg Oncol. 2018;25(8):2249–60. doi: 10.1245/s10434-018-6533-3 29855830 PMC6039971

[33] Alongi AM, Pettis J, Rivere A, Elder EA, Fuhrman G. Institutional analysis of metaplastic breast cancer. Am Surg. 2023;89(8):3579–81. doi: 10.1177/00031348231161691 36897265

[34] Cimino-Mathews A, Verma S, Figueroa-Magalhaes MC, Jeter SC, Zhang Z, Argani P, et al. A clinicopathologic analysis of 45 patients with metaplastic breast carcinoma. Am J Clin Pathol. 2016;145(3):365–72. 27124919 10.1093/ajcp/aqv097

[35] Tan Y, Yang B, Chen Y, Yan X. Outcomes of metaplastic breast cancer versus triple-negative breast cancer: a propensity score matching analysis. World J Surg. 2023;47(12):3192–202. doi: 10.1007/s00268-023-07106-1 37709983

[36] Rakha EA, Tan PH, Varga Z, Tse GM, Shaaban AM, Climent F, et al. Prognostic factors in metaplastic carcinoma of the breast: a multi-institutional study. Br J Cancer. 2015;112(2):283–9. doi: 10.1038/bjc.2014.592 25422911 PMC4453452

[37] Tadros AB, Sevilimedu V, Giri DD, Zabor EC, Morrow M, Plitas G. Survival outcomes for metaplastic breast cancer differ by histologic subtype. Ann Surg Oncol. 2021;28(8):4245–53. doi: 10.1245/s10434-020-09430-5 33389291

